# Spin Caloritronics in 3D Interconnected Nanowire Networks

**DOI:** 10.3390/nano10112092

**Published:** 2020-10-22

**Authors:** Tristan da Câmara Santa Clara Gomes, Nicolas Marchal, Flavio Abreu Araujo, Luc Piraux

**Affiliations:** Institute of Condensed Matter and Nanosciences, Université Catholique de Louvain, Place Croix du Sud 1, 1348 Louvain-la-Neuve, Belgium; tristan.dacamara@uclouvain.be (T.d.C.S.C.G.); nicolas.marchal@uclouvain.be (N.M.); flavio.abreuaraujo@uclouvain.be (F.A.A.)

**Keywords:** 3D nanowire networks, spin caloritronics, thermoelectricity, spintronics, giant magnetoresistance multilayers

## Abstract

Recently, interconnected nanowire networks have been found suitable as flexible macroscopic spin caloritronic devices. The 3D nanowire networks are fabricated by direct electrodeposition in track-etched polymer templates with crossed nano-channels. This technique allows the fabrication of crossed nanowires consisting of both homogeneous ferromagnetic metals and multilayer stack with successive layers of ferromagnetic and non-magnetic metals, with controlled morphology and material composition. The networks exhibit extremely high, magnetically modulated thermoelectric power factors. Moreover, large spin-dependent Seebeck coefficients were directly extracted from experimental measurements on multilayer nanowire networks. This work provides a simple and cost-effective way to fabricate large-scale flexible and shapeable thermoelectric devices exploiting the spin degree of freedom.

## 1. Introduction

Spin-dependent transport mechanisms are expected to play a crucial role in the development of next generation of thermoelectric devices [1]. Therefore, a central focus of the rapidly emerging field of spin caloritronics is combining heat-driven transport with spintronics [2,3]. Previous studies on nanoscale metal structures, magnetic tunnel junctions and magnetic insulators have led to the observation of various spin-enabled mechanisms that may differ significantly from conventional thermoelectrics effects, including spin Seebeck effects [4,5], thermally driven spin injection [6] and thermal assisted spin-transfer torque [7,8]. However, dimensions in magnetic nanostructures lead to major experimental issues such as insufficient power output capability and lack of reliable methods to obtain key spin caloritronic parameters, and have limited the application of spin caloritronic devices based on these effects [9,10].

In this context, recently developed interconnected magnetic nanowire (NW) networks embedded within porous polymer films provide a simple and cost-effective pathway to fabricate flexible and shapeable, macroscopic-scale spintronic nanoarchitectures with advantageous thermoelectric properties. They combine the macroscopic dimension required for large thermoelectric power output with the nanostructuration required to enable spintronic effects, and meet the mechanical, electrical and thermal stability required for practical applications. Indeed, ferromagnetic transition metals exhibit large diffusion thermopowers because of the pronounced structure of the d-band and the high energy derivative of the density of states at the Fermi level [11], while they also exhibit significant magnon-drag contribution to the thermoelectric power within a wide temperature range [11,12], leading to both positive and negative large Seebeck coefficient *S* at room temperature (RT). Moreover, due to their large electrical conductivity σ, they can exhibit very large power factors $\mathrm{PF}=S^{2}\sigma$, which is the physical parameter that relates to the output power density of a thermoelectric material, that remain in interconnected NW structures made of ferromagnetic metals. Therefore, interconnected magnetic NW networks allow to obtain both p-type and n-type light, robust, flexible and shapeable thermoelectric elements, which are both required for practical thermometric devices. Besides, interconnected networks made of multilayered NWs, with a succession of ferromagnetic metal (FM) and normal metal (NM) layers, allow for large magnetic control of thermoelectric transport and for the precise and direct extraction of spin-dependent Seebeck coefficients from experimental measurements. Therefore, such NW-based spin caloritronics devices overcome the insufficient power generation capability inherent to the custom-patterned nanoscale magnetic structures reported previously and constitute promising candidates for heat management applications [13,14].

## 2. Materials and Methods

**Figure 1:**
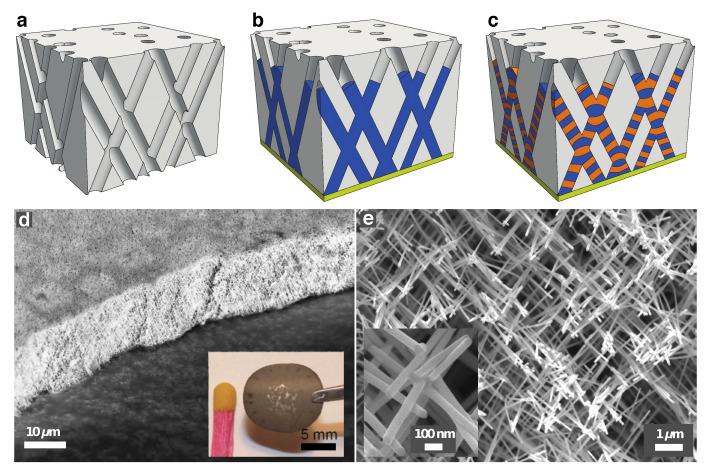
(**a**) Schematics of the 3D nanoporous polymer template, (**b**) crossed nanowire and (**c**) crossed nanowire network with alternating magnetic and non-magnetic layers. (**d**,**e**) SEM images of self-supported interconnected nanowire network with different magnifications. (**d**) Low-magnification image showing the 50${}^{\circ }$ tilted view of a macroscopic nanowire network film with 105 nm diameter and ∼20% packing density. The inset displays an optical image showing the size and mechanical robustness of the macroscopic self-supporting network. (**e**) Low magnification image showing the top view of the NW network with 80 nm diameter and ∼3% packing density. The inset shows the nanowire branched structure at higher magnification.

Three-dimensional (3D) nanoporous templates were obtained by a track-etched technique with a sequential multi-step exposure of energetic heavy ions, at various angles with respect to the normal of polycarbonate (PC) film surface [15,16]. It allows to obtain 20 μm thick template films with distinct porosities and pores sizes, as illustrated in Figure 1a. In the present study, the as-prepared polymer membranes containing networks of interconnected cylindrical nanopores were designed with pores of well-defined diameter of about 80 nm and with porosity P≈ 3%. Then, the PC membranes were filled with 3D metallic NWs by direct electrodeposition at RT in the potentiostatic mode using a Ag/AgCl reference electrode and a Pt counter electrode, which allows for a very high degree of replication of the nanopores [16,17,18]. For this, the PC templates were coated on one side with a Au/Cr bilayer using an e-beam evaporator to serve as cathode during the electrochemical deposition. Interconnected homogeneous NW networks, as illustrated in Figure 1b, made of several ferromagnetic pure metals (Ni, Co) and alloys (NiFe, CoNi, NiCr, CoCr) were grown by electrodeposition from home-made electrolyte solutions with fine-tuned metallic ions concentration and pH acidity at calibrated constant potentials [16,17,18,19,20]. In addition, FM and Cu layers were electrochemically stacked in the host 3D porous templates to make interconnected FM/Cu multilayered NW networks, with FM = Co, Co${}_{50}$Ni${}_{50}$, Ni${}_{80}$Fe${}_{20}$ and Ni, as illustrated in Figure 1c. They were grown by electrodeposition from single home-made electrolyte solution using a pulsed electrodeposition technique [13,14,21,22].

**Figure 2:**
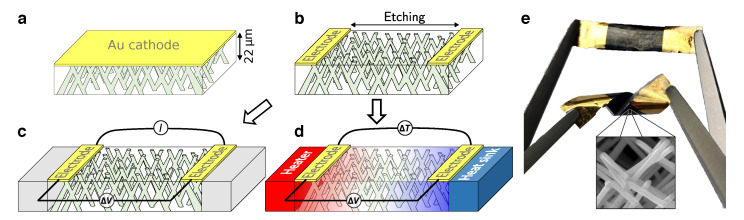
3D interconnected nanowire networks and experimental set-ups for measurement of transport properties. (**a**) Schematic of 3D interconnected nanowire network film grown by electrodeposition from a Au cathode into a 20 μm thick polycarbonate template with crossed-nanopores. (**b**) Two-probe electrodes design obtained by local etching of the Au cathode. (**c**,**d**) Device configuration for successive measurements of the resistance and the Seebeck coefficient. (**c**) The voltage differential ΔV induced by the injected current *I* between the two metallic electrodes is measured while the two electrodes are maintained at an identical and constant temperature. (**d**) Heat flow is generated by a resistive element at one electrode while the other electrode is maintained at desired temperature. The temperature difference ΔT between the two metallic electrodes is measured by a thermocouple while thermoelectric voltage ΔV settles. (**e**) Photograph of a flexible device made of 3D interconnected nanowires embedded in a polycarbonate matrix and with the two gold electrodes design. The inset SEM image shows the nanowire branched structure with diameter of 80 nm and ∼3% packing density.

The interconnected NW structure was characterized using a field-emission scanning electron microscope (FE-SEM) after complete chemical dissolution of the cathode and the template. The crossed NW networks are mechanically robust and self-standing, as shown by Figure 1d,e where SEM images of the networks are presented. As seen, the NW networks exhibit the replicated complex branching morphology of the porous template. The inset in Figure 1d shows the typical size as well as the mechanical robustness of the macroscopic self-supporting networks. In order to perform magnetoresistance (MR) and magneto-thermoelectric power (MTP) measurements on the interconnected NWs embedded into the PC film (schematically shown in Figure 2a), the Au cathode was locally etched by plasma to pattern two Au electrodes with low contact resistance, as illustrated in Figure 2b–d. The Seebeck coefficient was measured by inducing a temperature difference ΔT using a resistive element and measuring the induced thermoelectric voltage ΔV, following the procedure described in refs. [13,22]. MR and MTP were measured by applying an external magnetic field along the out-of-plane (OOP) and in-plane (IP) directions of the NW network films, with a maximum applied field of ±10 kOe. The measurements were made at temperatures from 10 K to 320 K. Other benefits of the 3D interconnected nanowire network system are its light weight and flexibility. For instance, the density of Co NWs fully filling a PC membrane was estimated to be about 1.4 g/cm${}^{3}$. The flexibility of the network films is shown in Figure 2e. As seen, the film can be easily twisted without damaging its electrical properties. The electrical resistance was measured during several successive torsions of a network of interconnected nanowires, revealing only a marginal variation in resistance, less than 0.2%.

## 3. Results and Discussion

### 3.1. Homogeneous Nanowire Networks

The resistance and Seebeck coefficient of homogeneous NW networks were measured for T= 10–320 K. Table 1 provides the Seebeck coefficients of homogeneous NW networks made of several metals and alloys at RT. Both n-type and p-type thermoelectric NW networks with large absolute thermopower values were obtained. The results are consistent with reported bulk Seebeck coefficients [23,24,25,26]. Moreover, the value reported for the Co${}_{50}$Ni${}_{50}$ NW network is also consistent with the ones reported for parallel arrays of NiCo NWs [27,28]. The resistivity of the homogeneous NW networks were estimated assuming that the Matthiesen’s rule holds for the different metallic NW networks. In that case, the resistivity at RT is given by $\rho_{\mathrm{NWs}}^{\mathrm{RT}}=\rho_{\mathrm{FM}}^{\mathrm{RT}}+\rho_{\mathrm{NWs}}^{0}$, where $\rho_{\mathrm{FM}}^{\mathrm{RT}}$ is the resistivity of the FM that composes the NWs at RT due to thermally excited scatterings and $\rho_{\mathrm{NWs}}^{0}$ is the residual resistivity of the NWs due to impurities along with surface scattering within the NW network and internal grain-boundary scattering. For NW diameter not too small (ϕ≥ 40 nm), the thermally induced scattering effects are independent on the sample dimensions, nanostructuration and defect concentration [29]. Therefore, $\rho_{\mathrm{FM}}^{\mathrm{RT}}$ can be taken as the ideal resistivity values at RT reported for bulk materials in the literature (from refs. [30,31,32,33]). Moreover, because the resistivity due to thermally excited scatterings tends to 0 at low temperatures, the resistivity at 10 K of the NW networks can be approximated to $\rho_{\mathrm{NWs}}^{10}\;K\approx \rho_{\mathrm{NWs}}^{0}$. Finally, using the residual resistivity ratio $\mathrm{RRR}=R_{\mathrm{NWs}}^{\mathrm{RT}}/R_{\mathrm{NWs}}^{10}\;K\approx \left(\rho_{\mathrm{FM}}^{\mathrm{RT}}+\rho_{\mathrm{NWs}}^{0}\right)/\rho_{\mathrm{NWs}}^{0}$, the RT resistivity of the NWs can be estimated as $\rho_{\mathrm{NWs}}^{\mathrm{RT}}\approx \rho_{\mathrm{FM}}^{\mathrm{RT}}\mathrm{RRR}/\left(\mathrm{RRR}-1\right)$. The calculated resistivity are provided in Table 1. Slightly larger resistivity compared to the bulk materials have been obtained, as expected for electrodeposited nanostructured materials. Indeed, electrodeposited materials display a relatively large amount of defects, leading to defect scattering into the NWs, while the NW transverse nanoscale dimensions lead to surface scattering effect. This engenders larger residual resistivity of the NW networks compared to bulk materials.

Table 1 also provides the power factors PF $=S^{2}/\rho$ together with the figure of merit ZT for the NW networks at RT. Due to the slightly larger electrical resistivity of the NW networks compared to bulk materials, slightly lower PF values have been found with respect to the bulk values. However, the PF values obtained are similar an even larger than to that of widely used thermoelectric material bismuth-telluride (in the range 1–6 mW/K${}^{2}$m) and at least one order of magnitude larger than the ones reported for flexible thermoelectric films based on optimized conducting polymers [34,35]. Large RT value of PF of about 11.0 mW/K${}^{2}$m have been obtained in interconnected Co NWs, which is almost as good as the bulk Co that display the largest PF value of about 15 mW/K${}^{2}$m [36]. Moreover, regarding p-type materials, the RT PF estimated for the Fe and Ni${}_{96}$Cr${}_{4}$ NW networks are close to the largest PF at RT of about 9 mW/K${}^{2}$m found for CePd${}_{3}$ [37,38]. Therefore, electrodeposited magnetic NW networks are suitable for both n-type and p-type thermoelectric modules, in particular for active cooling applications as it has been recently shown that such application requires materials exhibiting large PF [38,39]. The efficiency of a material’s thermoelectric energy conversion is determined by its figure of merit $ZT=S^{2}\sigma T/\kappa$, with κ the thermal conductivity. In a previous study, M. Ou et al [40] have measured the thermal conductivity of a suspended Ni NW for T= 15–300 K. While the Lorenz ratio L=κ/σT departs from the Sommerfeld value ($L_{0}=2.45\cdot 10^{-8}$ V${}^{2}$/K${}^{2}$) at low temperatures, *L* was found to be equal to $L_{0}$ with a 5% margin of error above T= 50 K. Due to the very low thermal conductivity of polycarbonate (κ= 0.2 W/Km at RT), the contribution of the polymer matrix to heat transport is much smaller than that of the metallic NW network. Indeed, assuming that the Wiedemann–Franz law holds for the NWs, estimations of the RT electronic thermal conductivities $\kappa_{e}=L_{0}T/\rho$ provides values between 10 and 100 W/Km, hence at least two orders of magnitude above the thermal conductivity of the PC template. Moreover, in the limits of the Wiedemann–Franz law, the ZT value can be approximated by $ZT\approx S^{2}/L_{0}$. Using this approximation, the ZT values of the NWs networks at RT have been estimated and are reported in Table 1. Although the figure of merit is more than one order of magnitude smaller than those of state-of-the-art thermoelectric materials (ZT≈ 1 in BiTe alloys), it is comparable to those of thermocouple alloys (ZT≈ 6 · 10${}^{-2}$ and ZT≈ 1.4 · 10${}^{-2}$ in constantan and chromel, respectively) and can be used in applications for devices with low energy requirements when the supply of heat essentially is free as with waste heat.

**Figure 3:**
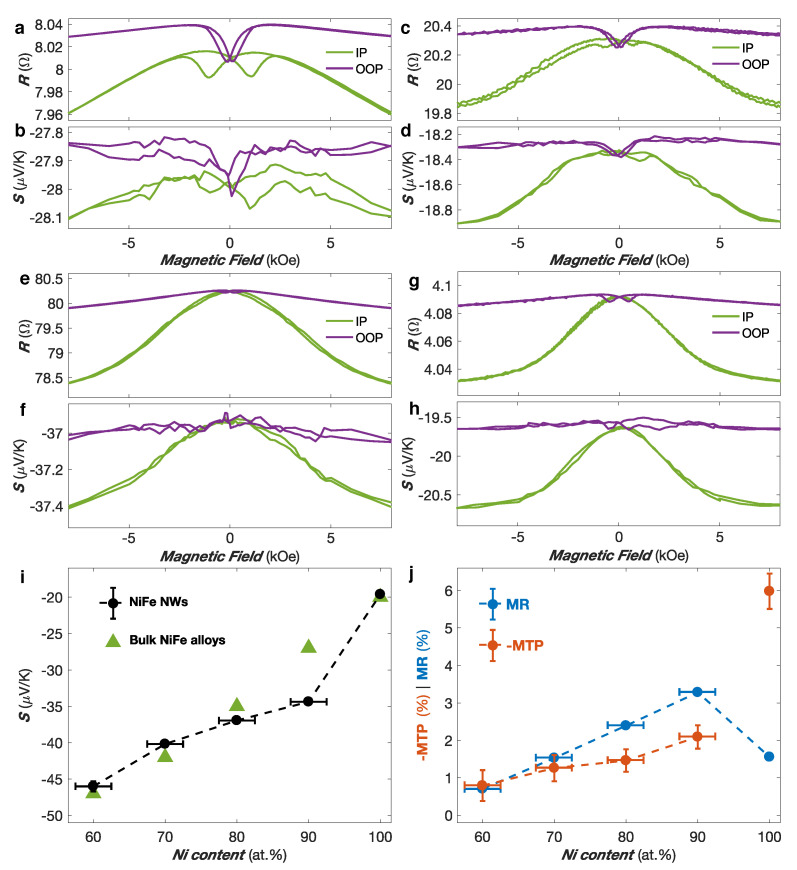
(**a**–**h**) Room-temperature variation of the electrical resistance and Seebeck coefficient of Co (**a**,**b**), Co${}_{50}$Ni${}_{50}$ (**c**,**d**), Ni${}_{80}$Fe${}_{20}$ (**e**,**f**) and Ni (**g**,**h**) nanowire networks obtained with the magnetic field applied along the in-plane (IP—green) and out-of-plane (OOP—purple) directions of the nanowire network film. (**i**) Variation of the Seebeck coefficient vs Ni content in NiFe nanowire networks at room temperature. Values previously reported for bulk alloys [41] are also shown. (**j**) Magnetoresistance (MR—blue) and magneto-thermopower (MTP—red) ratios as a function of Ni content in NiFe nanowire networks at room temperature.

Besides, magneto-transport measurements have been conducted. Figure 3a–h shows the variation of resistance and Seebeck coefficient with a magnetic field applied along the IP and OOP direction for the Co (a–b), Co${}_{50}$Ni${}_{50}$ (c–d), Ni${}_{80}$Fe${}_{20}$ (e–f) an Ni (g–h) NW networks. The MR curves show the anisotropic magnetoresistance (AMR) effect that leads to a decrease in resistivity as the angle between the magnetization and current directions is increased. Indeed, the current flow being restricted along the NW segments, the saturation magnetization in the IP direction makes an average angle of ±65${}^{\circ }$ with the current. By comparison, when the magnetization is saturated in the OOP direction, the average angle between the magnetization and the current directions is much smaller (±25${}^{\circ }$), leading to a larger resistance at saturation when the field is applied in the OOP direction than when the field is applied in the IP direction. The lower resistance state expected for the perpendicular configuration between magnetization and current could not be achieved in such NW networks due to their geometry. Figure 3a–h also shows that the absolute value of the thermopower increases with increasing angle between the magnetization and the current flow for the NW networks. At saturation, larger absolute thermopower is obtained in the IP direction compared to the OOP direction. This is in good agreement with previous studies performed on single NWs [27]. Moreover, as shown in Figure 3a–h for all NW networks, the MR and MTP effects exhibit similar behavior, indicating a direct relation between the two effects, as expected from Mott’s formula for the diffusion thermopower. Table 1 provides the RT values of the MR and MTP ratio, defined as MR $=\left(R_{\max}-R_{\min}\right)/R_{\max}$ and MTP $=\left(S_{\max}-S_{\min}\right)/S_{\max}$, respectively. Similar amplitudes have been obtained for the Co, Co${}_{50}$Ni${}_{50}$ and NiFe alloys networks. The slightly larger MTP ratio amplitude compared to the MR ratio amplitude observed in Co${}_{50}$Ni${}_{50}$ is also consistent with previous studies performed on NiCo alloy NWs [27,28]. In contrast, Figure 3g,h reveals an enhancement of the MTP ratio up to three times larger than the corresponding MR ratio amplitude for the Ni NW network, in spite of similar field dependencies. Such definition of the MR ratios leads to an underestimation of the traditional AMR ratio defined as $\left(R_{\|}-R_{⊥}\right)/R_{⊥}$, where $R_{\|}$ and $R_{⊥}$ are the theoretical resistance states obtained for parallel and perpendicular orientations of the magnetization and current directions, respectively. However, the AMR ratio can be extracted from the resistance states measured at saturation in the OOP and IP directions $R_{\mathrm{OOP}}$ and $R_{\mathrm{IP}}$ using the analytical model described in refs. [17,18,19].

Figure 3i shows the Seebeck coefficients *S* at RT of the NW networks made of Ni${}_{x}$Fe${}_{1-x}$ alloys with 0.6 ≤x≤ 1. As seen, the thermopower increases continuously with increasing Fe content, reaching values between −20 μV/K for pure Ni to about −45 μV/K for Ni${}_{60}$Fe${}_{40}$. These results are in good agreement with the experimental data obtained on bulk NiFe alloys [24,41]. Therefore, NiFe alloys with fine-tune composition potentially yield significantly larger Seebeck coefficients than pure ferromagnetic metals like Co and thermocouple materials like constantan (Cu${}_{55}$Ni${}_{45}$: S≈ −38 μV/K). The measured value for Ni${}_{80}$Fe${}_{20}$ NWs (S≈ −37 μV/K) is also very similar to the reported bulk values in the literature [25,26]. Figure 3j shows the magnitude of the MR and MTP ratios evaluated at RT for pure Ni and Ni${}_{x}$Fe${}_{1-x}$ alloy NW networks as a function of the Ni content *x*. It reveals a peak in the MR ratio for alloying compositions around 90% of Ni, in coherence with previous studies on bulk NiFe alloys [42,43]. Moreover, it highlights the very different behavior of the Ni NW network, compared to the NiFe alloys. For the Ni${}_{x}$Fe${}_{1-x}$ alloy samples with 0.6 ≤x≤ 0.9, the magnitude of the MTP ratio is either comparable or smaller to the MR ratio. The smaller value of the MTP ratio with respect to the corresponding MR ratio for the Ni${}_{80}$Fe${}_{20}$ NW network is in agreement with measurements performed on Ni${}_{80}$Fe${}_{20}$ thin films [44]. In contrast, the Ni NW network exhibits a MTP effect of −6% much larger than the MR ratio of ∼1.6%. This result is in good agreement with previous measurements performed on single Ni NWs and parallel arrays of Ni NWs, where MTP ratios were found up to 2.5–3 times larger than the corresponding MR ratios [27,28,45] and may be related to the spin-dependent Seebeck coefficients, $S_{↑}$ and $S_{↓}$, of opposite sign [46]. It is interesting to note that for Ni thin films, the observed anisotropic MTP has approximately the same magnitude than the anisotropic MR (∼1.5%) [44]. Further studies are needed to understand this unexpected enhanced MTP for Ni NWs.

**Figure 4:**
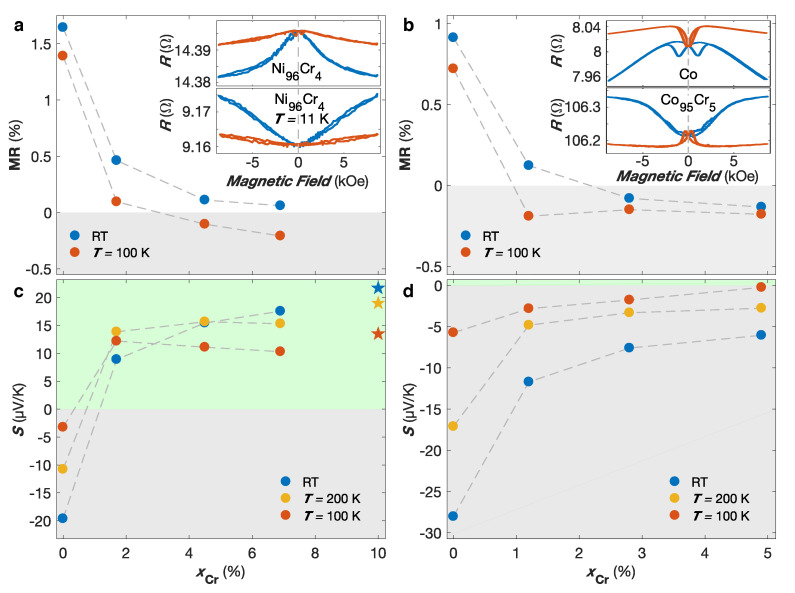
(**a**,**b**) Magnetoresistance (MR) ratio as a function of the Cr content for interconnected NiCr (**a**) and CoCr (**b**) nanowire networks at room temperature (RT—blue) and T= 100 K (red). The gray areas in (**a**,**b**) indicates negative anisotropic magnetoresistance. The inset in (**a**) compares the MR curves measured along the out-of-plane (red) and in-plane (blue) directions for the Ni${}_{96}$Cr${}_{4}$ crossed nanowire network at RT and T= 11 K. The inset in (**b**) compares the RT MR curves measured along the out-of-plane (red) and in-plane (blue) directions for the interconnected Co and Co${}_{95}$Cr${}_{5}$ nanowire networks. (**c**,**d**) Seebeck coefficient *S* at zero magnetic field as a function of the Cr content of for interconnected NiCr (**c**) and CoCr (**d**) nanowire networks at RT (blue), T= 200 K (yellow) and T= 100 K (red). The recommended values for chromel (Ni${}_{90}$Cr${}_{10}$) are indicated by star symbols. The green and gray areas in (**c**,**d**) indicate positive and negative *S* values, respectively. The symbol size encompasses the experimental error bars, which are set to two times the standard deviation of the experimental measurements of the electrical and temperature measurements, gathering 95% of the data variation.

The addition of transition metal impurities in ferromagnetic metals has been found to have a large influence on their spin-dependent electronic transport properties (see ref. [47] for a review). It can be ascribed to a matching/mismatching of the d-electronic states between the host ferromagnetic metal and the transition metal impurities [48]. Notably, diluted Cr impurities in Co, Fe and Ni generate stronger scattering in the majority-spin channel in a two-band model. This leads to a spin-asymmetry parameter $\alpha =\rho_{↓}/\rho_{↑}<1$, which means that minority spin electrons dominate the electronic conduction, contrasting to that of pure ferromagnetic metals. In this context, NW networks made of dilute NiCr and CoCr alloys (Cr content ≤ 7 at.%) have been fabricated, and the influence of impurity concentration on the AMR effect and thermopower has been investigated [20]. Figure 4a provides the MR ratio as a function of the Cr content for NiCr NW networks at RT and T= 100 K, where the MR ratio is defined as MR $=\left(R_{\mathrm{OOP}}-R_{\mathrm{IP}}\right)/R_{\mathrm{OOP}}$, with $R_{\mathrm{OOP}}$ and $R_{\mathrm{IP}}$ the resistance states reached at H= 10 kOe in the OOP and IP directions, respectively. Although this MR ratio underestimates the AMR effect, its sign correctly reflects the sign of the AMR effect. Indeed, when the magnetization is saturated along the OOP and IP directions, it makes respectively average angles of about 25${}^{\circ }$ and 65${}^{\circ }$ with the current flow that is strictly restricted along the NW segments. Therefore, the case $R_{\mathrm{OOP}}>R_{\mathrm{IP}}$ unambiguously indicates a positive AMR effect, as usually observed for ferromagnetic metals and alloys. As seen in Figure 4a, positive AMR have been found at RT for all interconnected NiCr NW networks, with a decrease of the MR ratio with increasing Cr content. Conversely, negative AMR effect at T= 100 K has been observed for NiCr NW networks with Cr content >4%. This is illustrated in the inset of Figure 4a that compares the MR curves measured at RT and T= 11 K for the Ni${}_{96}$Cr${}_{4}$ sample. While the AMR is positive at RT, the Ni${}_{96}$Cr${}_{4}$ NW network exhibits $R_{\mathrm{IP}}>R_{\mathrm{OOP}}$ at T= 11 K. Similar results have been observed on the NiCr NWs samples with Cr content > 4%. These observations are in agreement with previous negative AMR measurements reported at low temperatures in bulk NiCr dilute alloys [49,50,51].

Figure 4b shows the MR curves for the dilute CoCr NW networks, where negative AMR have been observed at RT for Cr concentrations ≥3 at.% contrasting to pure Co NWs [20]. Indeed, as seen in the inset of Figure 4b, the RT MR curves measured for the Co and Co${}_{95}$Cr${}_{5}$ samples show opposite behaviors ($R_{\mathrm{OOP}}>R_{\mathrm{IP}}$ and $R_{\mathrm{IP}}>R_{\mathrm{OOP}}$ for Co and Co${}_{95}$Cr${}_{5}$ NW networks, respectively). Moreover, all CoCr NWs exhibit negative AMR with similar amplitude for all alloying composition down to ∼1 at.%. Even if the interpretation is still controversial [52], Campbell, Fert and Jaoul have proposed a model based on a spin-orbit mechanism [50,53] predicting that the AMR ratio is proportional to (α−1) at low temperatures, where $\alpha =\rho_{↓}^{0}/\rho_{↑}^{0}$ is the spin asymmetry coefficient with $\rho_{↓}^{0}$ and $\rho_{↑}^{0}$ the residual resistivities of the spin down and spin up electrons. When α<1, negative AMR is thus expected. Moreover, in this simplified model, α is constant for a given impurity, independently of its concentration. When the temperature increases, the electron-phonon and electron-magnon scattering processes induces additional terms in the electrical resistivity. In consequence, as temperature rises, the AMR ratio of the dilute alloy should tend to that of the host pure ferromagnetic metal, converging more rapidly as the impurity concentration is lower [53]. The predictions of Campbell, Fert and Jaoul [50,53] are mostly in qualitatively good agreement with the experimental results obtained for both interconnected NiCr and CoCr NW networks and the theoretic prediction of spin asymmetry coefficient α<1 in these dilute alloys [47,54]. Notably, the negative MR ratios observed at low temperatures for CoCr NW networks are found to be independent of the Cr content, while the change of sign at RT is favored for the lowest Cr concentration (see Figure 4b). However, the fact that the MR ratio remains negative for Cr content ≥3 at.% in CoCr NWs is unexpected because thermally activated scattering of conduction electrons should significantly weaken the effect. Similar unexpected negative AMR ratios at RT have already been observed in earlier studies performed on bulk CoIr alloys [52]. One hypothetical justification for the RT negative MR ratio in the CoCr NW networks is that the residual resistivity due to impurity scatterings which is proportional to the Cr content becomes dominant compared to the thermally activated resistivity of the matrix at RT in CoCr alloys with Cr content ≥3%. Thus their influence over the AMR effect remain dominant at RT. This is illustrated by the residual ratio (R${}_{300\;K}$/R${}_{10\;K}$) below 2 (1.7 and 1.6 for 3 and 5 at.%Cr respectively) for the samples with RT negative AMR, up to 2 for the sample with positive RT AMR (5.4 and 2.7 for the Co and Co${}_{99}$Cr${}_{1}$ samples, respectively).

Figure 4c,d show the Seebeck coefficient as a function of the Cr concentration in the absence of any external magnetic field for the interconnected NiCr and CoCr NW networks at RT, T= 200 K and T= 100 K. As seen in Figure 4c, the thermopower of NiCr NWs abruptly changes sign, going from the pure Ni value (−20 μV/K) to about +17.5 μV/K for the Ni${}_{93}$Cr${}_{7}$ NW network [20]. Similar changes of sign in the Seebeck coefficient have been previously observed in bulk NiCr alloys with the electrical conduction dominated by minority spin electrons and have been related to drastic modifications in the density of states at the Fermi level by the addition of Cr impurities to the host ferromagnetic metals [46,47,55]. Figure 4c also provides the recommended values for the thermocouple material Chromel P (Ni${}_{90}$Cr${}_{10}$), which are consistent with the Seebeck coefficients reported for the studied samples. In contrast, the sign of the Seebeck coefficient does not change for any value of Cr concentration for CoCr NW networks, as seen in Figure 4d. The RT thermopower drops rapidly from −28 μV/K for pure Co to much smaller negative values approaching −5 μV/K for Co${}_{95}$Cr${}_{5}$ NWs. Similar features are also observed at lower temperatures. These observations are consistent with experiments previously conducted on dilute Co-based alloys with α<1 [11]. The sudden drop of *S* induced by the incorporation of few at. % of Cr in Co NWs can also be ascribed to significant changes in the density of states for the majority spin electrons. Please note that extremely small MTP effects (typically less than 1%) were found in all NiCr and CoCr samples.

### 3.2. Multilayered Nanowire Networks

We will now turn to the discussion to interconnected FM/Cu multilayered NW networks, which have been found to be good candidates for spin caloritronic devices with large spin-dependent thermoelectric transport properties as they combine large giant magnetoresistance (GMR) effect measured in the current-perpendicular-to-plane (CPP) configuration with large thermoelectric power factors [13,14,22]. In addition, their macroscopic dimension leads to efficient energy conversion and opens the door to practical applications. In this context, interconnected FM/Cu multilayered NW networks, with FM = Co, Co${}_{50}$Ni${}_{50}$ or Ni${}_{80}$Fe${}_{20}$ and Ni, have been investigated. In CPP FM/Cu multilayers, the Seebeck coefficient along the axial direction (perpendicular direction to the layers) can be calculated from the corresponding transport properties using Kirchhoff’s rules [56] as

(1) $$S_{⊥}=\frac{S_{\mathrm{Cu}}\kappa_{\mathrm{FM}}+\lambda S_{\mathrm{FM}}\kappa_{\mathrm{Cu}}}{\lambda \kappa_{\mathrm{Cu}}+\kappa_{\mathrm{FM}}}.$$

Here $S_{\mathrm{FM}}$, $S_{\mathrm{Cu}}$ and $\kappa_{\mathrm{FM}}$, $\kappa_{\mathrm{Cu}}$ represent the thermopower and the thermal conductivity of the FM and Cu and $\lambda =t_{\mathrm{FM}}/t_{\mathrm{Cu}}$ the thickness ratio of FM and Cu layers. If the Wiedemann–Franz law holds for the Cu and FM, Equation (1) simply reduces to

(2) $$S_{⊥}=\frac{S_{\mathrm{Cu}}\rho_{\mathrm{Cu}}+\lambda S_{\mathrm{FM}}\rho_{\mathrm{FM}}}{\lambda \rho_{\mathrm{FM}}+\rho_{\mathrm{Cu}}},$$

with $\rho_{\mathrm{FM}}$ and $\rho_{\mathrm{Cu}}$ being the corresponding electrical resistivities. According to Equation (2), in the limits $S_{\mathrm{FM}}\rho_{\mathrm{FM}}\gg S_{\mathrm{Cu}}\rho_{\mathrm{Cu}}$, which is usually acceptable, and a thickness ratio λ not too small, $S_{\mathrm{FM}/\mathrm{Cu}}$ is mainly determined by the large thermopower of the ferromagnetic metal. The same conclusion can be drawn when the FM layers are made of ferromagnetic alloys such as Co${}_{50}$Ni${}_{50}$ and Ni${}_{80}$Fe${}_{20}$, because of highly contrasting thermal conductivity values between these alloys and Cu. By comparison, the Seebeck coefficient of a planar FM/Cu multilayer stack in the direction parallel to the layers (CIP) is given by

(3) $$S_{\|}=\frac{S_{\mathrm{Cu}}\rho_{\mathrm{FM}}+\lambda S_{\mathrm{FM}}\rho_{\mathrm{Cu}}}{\lambda \rho_{\mathrm{Cu}}+\rho_{\mathrm{FM}}}.$$

**Figure 5:**
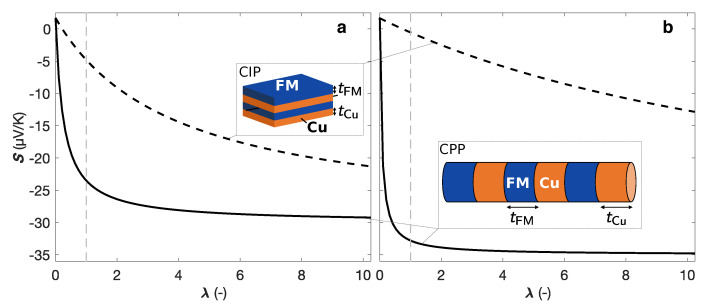
Calculated thermopowers for (**a**) Co/Cu and (**b**) Ni${}_{80}$Fe${}_{20}$/Cu multilayers in the layer parallel (dash-doted line—CIP) and perpendicular (solid line—CPP) directions as a function of the thickness ratio $\lambda =t_{\mathrm{FM}}/t_{\mathrm{Cu}}$, using Equations (2) and (3) and the bulk values for $S_{\mathrm{FM}}$, $\rho_{\mathrm{FM}}$, $S_{\mathrm{Cu}}$ and $\rho_{\mathrm{Cu}}$, with (**a**) FM = Co and (**b**) FM = Ni${}_{80}$Fe${}_{20}$. The grey dashed line shows the values for λ= 1. The insets in (**a**,**b**) show FM/Cu multilayer stacks with CIP (**a**) and CPP (**b**) configurations.

It shows that large thermopowers can be obtained only if the thickness ratio λ is very large, although in CIP system with dominant interface scattering, the thermopower becomes less sensitive to the Cu bulk resistance, which leads to larger *S* value [57]. This contrasting behavior between the Seebeck coefficient in the CIP and CPP configurations is illustrated in Figure 5 for Co/Cu (a) and Ni${}_{80}$Fe${}_{20}$/Cu (b) multilayers, using Equations (2) and (3) and literature room-temperature resistivity and thermopower values for bulk Co, Ni${}_{80}$Fe${}_{20}$ and Cu [23,25,30,41,58]. Although the electrical resistivity and thermal conductivity values for multilayered nanowires may vary significantly compared with their respective bulk constituents, the same trends in the contrasting behavior between the two configurations remain. So, CPP FM/Cu multilayers are promising candidates for good thermoelectric materials.

**Figure 6:**
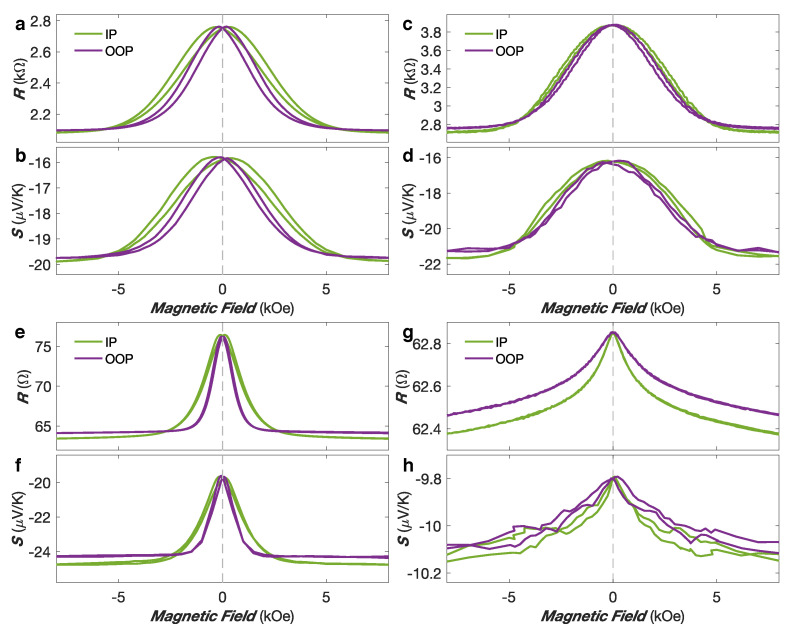
(**a**–**h**) Room-temperature variation of the electrical resistance and Seebeck coefficient of Co/Cu (**a**,**b**), Co${}_{50}$Ni${}_{50}$/Cu (**c**,**d**), Ni${}_{80}$Fe${}_{20}$/Cu (**e**,**f**) and Ni/Cu (**g**,**h**) multilayered nanowire samples obtained with the applied field in-plane (IP—green) and out-of-plane (OOP—purple) of the NW network film.

Figure 6 shows the resistance and thermopower variation with a magnetic field for the FM/Cu NW samples, with FM = Co (a–b), Co${}_{50}$Ni${}_{50}$ (c–d), Ni${}_{80}$Fe${}_{20}$ (e–f) and Ni (g–h). As shown in Figure 6a,b, the resistance and thermopower of the Co/Cu NW sample show the same magnetic field dependencies and similar relative changes of ∼25% at H= ±8 kOe at RT. The value of *S* in the saturated state of the Co/Cu NWs of about −20 μV/K is only slightly lower than the one reported for homogeneous Co NW networks (∼−28 μV/K), in good agreement with Equation (2) and Figure 5a. In addition, the sample is nearly magnetically isotropic, as observed from the magneto-transport curves obtained with the applied magnetic field along the OOP and IP directions. This behaviour corresponds to the one expected considering the crossed NW architecture and magneto-static arguments when using similar FM and NM layer thicknesses [59]. Similar features have been observed on the Co${}_{50}$Ni${}_{50}$/Cu and Ni${}_{80}$Fe${}_{20}$/Cu NW networks. As seen in Figure 6c,d, the resistance and thermopower of the Co${}_{50}$Ni${}_{50}$/Cu sample show the same magnetic field dependencies and relative changes of ∼30% at H= ±8 kOe at RT. Slightly higher Seebeck coefficients have been recorded in the Co${}_{50}$Ni${}_{50}$/Cu NW network, with a Seebeck coefficient in the saturated state in the IP direction of about −22 μV/K. These values are in agreement with the results obtained from measurements carried out on single Co${}_{50}$Ni${}_{50}$ and Co${}_{50}$Ni${}_{50}$/Cu NWs [27,60]. Figure 6e,f shows the RT resistance and thermopower of the Ni${}_{80}$Fe${}_{20}$/Cu NW network. Despite very similar magnetic field dependencies, the relative changes in Seebeck coefficient (∼26%) is found to be larger than the one of the resistance (∼17%) at H= ±8 kOe. As expected, the measured RT thermopower on the CPP-GMR Ni${}_{80}$Fe${}_{20}$/Cu sample in the saturated state (∼−25 μV/K) is only slightly smaller than the value found in the homogeneous Ni${}_{80}$Fe${}_{20}$ NW network (∼−35 μV/K), in good agreement with Equation (1) and Figure 5b. In contrast, the RT Seebeck coefficients reported for Ni${}_{80}$Fe${}_{20}$/Cu multilayers in the CIP geometry (∼−10 μV/K) are much smaller [61]. A contrasting behavior has been observed in interconnected Ni/Cu NW networks. Figure 6g,h shows the RT resistance and thermopower field dependencies with the applied magnetic field along the IP and OOP directions of the Ni/Cu NW network. Despite similar field dependencies, the amplitude of the magneto-thermopower effect (MTP ≈ −3.7%) is about four times larger than the corresponding magnetoresistance effect (MR ≈ 0.8%) at RT. This larger value of the MTP with respect to the MR ratio is consistent with our result obtained in homogeneous Ni NWs and previous studies on Ni/Cu multilayers [62]. This significant boost of the MTP, compared to MR may indicate underlying contrasting $S_{↑}$ and $S_{↓}$ values. This is consistent with the values of $S_{↑}=$ −43 μV/K and $S_{↓}=$ 20 μV/K reported by Cadeville and Roussell [46].

Table 2 provides the Seebeck coefficient in the saturated states ($S_{P}$) for the different FM/Cu NW network together with the estimated electrical resistivity ($\rho_{P}$) and the thermoelectric power factor (${\mathrm{PF}}_{P}$) at RT (see [13,14] for more details about the resistivity calculation). It shows that the CPP geometry of the device is suitable for spin caloritronic purposes since the RT thermopower and power factor of the FM/Cu NW networks in the saturated state are only slightly smaller than the value found in the pure corresponding ferromagnetic materials, as expected from conventional rules for a stacking arrangement in series (see Equation (2)). The figure of merit of the FM/Cu NW networks was estimated by $ZT=S^{2}/L_{0}$ at RT [13,14] and is reported in the saturated state in Table 2. The values found are comparable to those of homogeneous NW networks. Furthermore, considering the energy conversion from heat to electric power, the ZT of the proposed device is much larger than that of a spin Seebeck power generator (ZT≈ 10${}^{-4}$) based on a device using a two-step conversion process and the inverse spin Hall effect to convert spin current to charge current in non-magnetic materials [63]. Finally, Table 2 also provides the values at RT of the magnetoresistance ratio $\mathrm{MR}=\left(R_{\mathrm{AP}}-R_{P}\right)/R_{\mathrm{AP}}$, with $R_{\mathrm{AP}}$ and $R_{P}$ being respectively the high- and low-resistance states, and the absolute value of the magneto-thermopower $\mathrm{MTP}=\left(S_{\mathrm{AP}}-S_{P}\right)/S_{\mathrm{AP}}$, with $S_{\mathrm{AP}}$ and $S_{P}$ the corresponding thermopowers in the high- and low-resistance states, respectively, for the FM/Cu NW networks.

**Figure 7:**
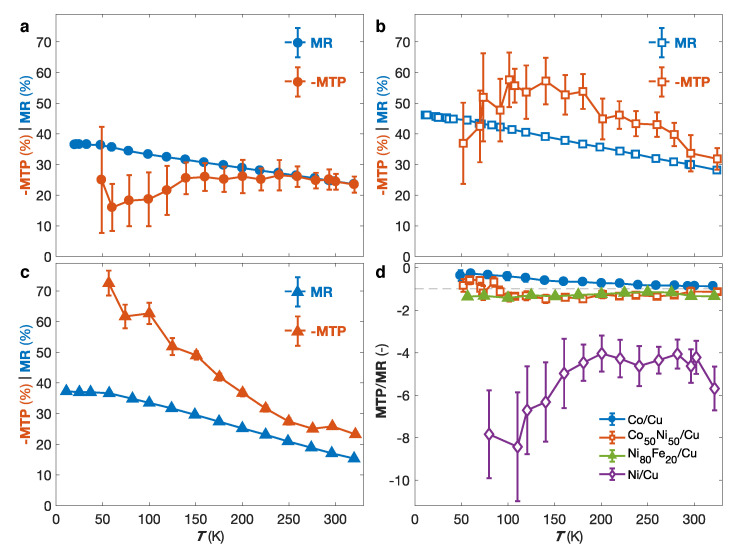
(**a**–**c**) MR and −MTP values as a function of temperature with the field applied in the plane of the (**a**) Co/Cu, (**b**) Co${}_{50}$Ni${}_{50}$/Cu and (**c**) Ni${}_{80}$Fe${}_{20}$/Cu nanowire network films. (**d**) Temperature dependencies of the ratio MTP/MR obtained in IP for the samples in (**a**–**c**) compared to the Ni/Cu nanowire network. The error bars in (**a**–**d**) reflect the uncertainty of the electrical and temperature measurements and is set to two times the standard deviation, gathering 95% of the data variation.

Figure 7a–c shows the temperature dependence of the MR and MTP ratios for the Co/Cu (a), Co${}_{50}$Ni${}_{50}$/Cu (b) and Ni${}_{80}$Fe${}_{20}$/Cu (c) NW networks. As seen, in all samples, the MR ratio shows a monotonic increase before reaching a plateau at low temperatures. This is expected because of the saturation of the resistivity at low temperatures and the vanishing of the spin mixing effect. Besides, for all three samples, the value of −MTP shows a similar increase with decreasing temperature as the MR ratio in the temperature range near RT. In contrast, in the low temperature range, the MTP exhibits very different behaviors depending on the material considered. For the Co/Cu sample, the MTP exhibits a less pronounced effect compared to the MR ratio, which is consistent with previous temperature dependency measurements of the MTP for Co/Cu multilayered NW networks [64]. For the Co${}_{50}$Ni${}_{50}$/Cu sample, similar MR and −MTP values are observed over the whole investigated temperature range, while for the Ni${}_{80}$Fe${}_{20}$/Cu sample, the MTP exhibits a pronounced reinforcement compare to the MR in the low temperature range. Figure 7d shows the ratio between the MR and MTP of the different FM/Cu NW networks, with FM = Co, Co${}_{50}$Ni${}_{50}$, Ni${}_{80}$Fe${}_{20}$ and Ni, for temperatures in the range of 50 K to 320 K. While the ratios for Co/Cu, Co${}_{50}$Ni${}_{50}$/Cu and Ni${}_{80}$Fe${}_{20}$/Cu remain below −1.5, the MTP/MR values of the Ni/Cu sample are encompassed between −4 and −8 in the whole temperature range, revealing an enhanced MTP effect with respect to the corresponding MR effect in Ni/Cu multilayered NW networks. A MTP ratio of about −14% was obtained at T= 80 K for a corresponding MR slightly below 2% for the Ni/Cu NW network. Such larger value of the MTP with respect to the MR ratio is consistent with our results in Ni homogeneous NW networks, and previous studies in Ni/Cu multilayers [62], and may be ascribed to Seebeck coefficients for spin up and spin down electrons of opposite sign. Furthermore, the magneto-power factor ($\mathrm{MPF}=\left({\mathrm{PF}}_{P}-{\mathrm{PF}}_{\mathrm{AP}}\right)/{\mathrm{PF}}_{\mathrm{AP}}$) can be expressed as $\mathrm{MPF}={\left(1-\mathrm{MTP}\right)}^{2}/\left(1-\mathrm{MR}\right)-1$. This yields RT MPF ratio of 111%, 155%, 92% and 8% for the Co/Cu, Co${}_{50}$Ni${}_{50}$/Cu, Ni${}_{80}$Fe${}_{20}$/Cu and Ni/Cu NW networks, respectively.

**Figure 8:**
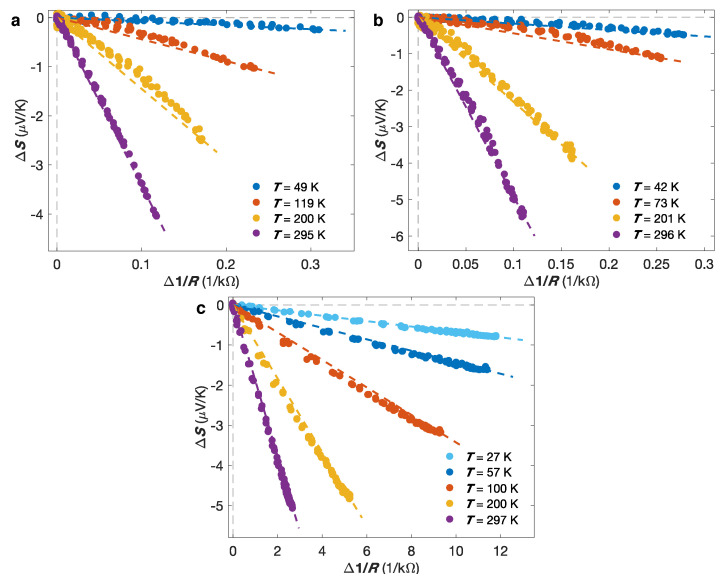
Linear variation of $\Delta S\left(H\right)=S\left(H\right)-S_{\mathrm{AP}}$ vs. $\Delta \left(1/R\left(H\right)\right)=1/R\left(H\right)-1/R_{\mathrm{AP}}$ at different measured temperatures, illustrating the Gorter-Nordheim characteristics for the (**a**) Co/Cu, (**b**) Co${}_{50}$Ni${}_{50}$/Cu and (**c**) Ni${}_{80}$Fe${}_{20}$/Cu nanowire networks. The solid lines correspond to the theoretical relation shown in Equation (4).

Figure 8a–c present evidences that the thermopower is dominated by electron diffusion over the whole temperature range investigated for the different multilayered NW networks considered. Defining the diffusion thermopower $S\left(H\right)=eL_{0}T\rho^{\prime }\left(H\right)/\rho \left(H\right)$ by Mott’s formula with $\rho^{\prime }\left(H\right)={\left(d\rho \left(H\right)/d\epsilon \right)}_{\epsilon =\epsilon_{F}}$ the derivative of the electrical resistivity with respect to the energy evaluated at the Fermi level $\epsilon_{F}$, the diffusion thermopower for antiparallel (AP) and parallel (P) arrangement of the successive FM layer magnetization can be written as $S_{\mathrm{AP}}=eL_{0}T\rho_{\mathrm{AP}}^{\prime }/\rho_{\mathrm{AP}}$ and $S_{P}=eL_{0}T\rho_{P}^{\prime }/\rho_{P}$. Then, the following expression describing an inverse relationship between the field-dependent thermopower S(H) and electrical resistance R(H) can be obtained [21,57,65]:

(4) $$S\left(H\right)=A+\frac{B}{R(H)},$$

where $A=\left(S_{\mathrm{AP}}R_{\mathrm{AP}}-S_{P}R_{P}\right)/\left(R_{\mathrm{AP}}-R_{P}\right)$ and $B=R_{\mathrm{AP}}R_{P}\left(S_{P}-S_{\mathrm{AP}}\right)/\left(R_{\mathrm{AP}}-R_{P}\right)$. This expression corresponds to an equivalent form of the Gorter-Nordheim relation for diffusion thermopower in metals and alloys [11], and has been observed at different temperatures in the FM/Cu NW network. This is illustrated by Figure 8a–c, which display $\Delta S\left(H\right)=S\left(H\right)-S_{\mathrm{AP}}$ with respect to $\Delta \left(1/R\left(H\right)\right)=1/R\left(H\right)-1/R_{\mathrm{AP}}$ for the (a) Co/Cu, (b) Co${}_{50}$Ni${}_{50}$/Cu and (c) Ni${}_{80}$Fe${}_{20}$/Cu samples. The dashed lines correspond to the theoretical linear relation ΔS(H)=BΔ(1/R(H)). The data show relatively good accordance with the theoretical linear variation, despite some slight deviation, in particular in the intermediate temperature range.

**Figure 9:**
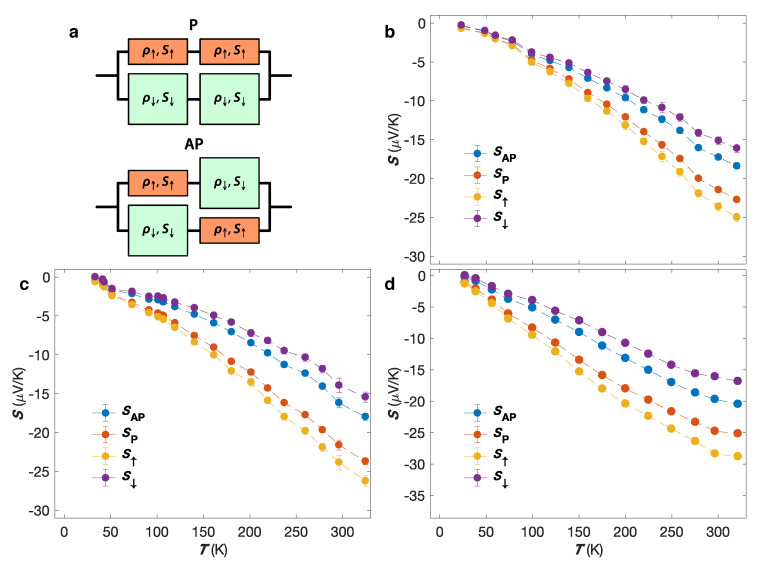
(**a**) The two-current model for the resistivity and the thermopower considering both parallel (P) and antiparallel (AP) magnetic configurations. (**b**–**d**) Measured Seebeck coefficients at zero applied fields $S_{\mathrm{AP}}$ (blue circles) and at saturating magnetic fields $S_{P}$ (red circles) of interconnected (**b**) Co/Cu, (**c**) Co${}_{50}$Ni${}_{50}$/Cu and (**d**) Ni${}_{80}$Fe${}_{20}$/Cu nanowire networks, along with the corresponding calculated spin-dependent Seebeck coefficients $S_{↑}$ (orange circles) and $S_{↓}$ (violet circles) using Equations (9) and (10). The error bars reflect the uncertainty of the electrical and temperature measurements and is set to two times the standard deviation, gathering 95% of the data variation.

In the limits of no-spin relaxation, most of the CPP-GMR data can be understood using a simple two-current series-resistor model, in which the resistance of layers and interfaces simply add and where ’up’ and ’down’ charge carriers are propagating independently in two spin channels with large spin asymmetries of the electron’s scattering [66,67]. Similarly, significantly different Seebeck coefficients for spin-up and spin-down electrons, $S_{↑}$ and $S_{↓}$, are expected because the d-band is exchange-split in these ferromagnets, as suggested from previous studies performed on dilute magnetic alloys [46,55]. Assuming that the layers of the magnetic multilayers are thin compared to the spin-diffusion lengths and according to the usual rule when the currents split to flow along two parallel paths (See Figure 9a), the corresponding thermopowers $S_{\mathrm{AP}}$ and $S_{P}$ are simply given by [57]

(5) $$S_{\mathrm{AP}}=\frac{S_{↑}\rho_{↑}+S_{↓}\rho_{↓}}{\rho_{↑}+\rho_{↓}}$$

and

(6) $$S_{P}=\frac{S_{↑}\rho_{↓}+S_{↓}\rho_{↑}}{\rho_{↑}+\rho_{↓}},$$

where separate resistivities $\rho_{↑}$ and $\rho_{↓}$ and Seebeck coefficients $S_{↑}$ and $S_{↓}$ are defined for majority and minority spin channels. From Equations (5) and (6), the following relations can be extracted:

(7) $$S_{\mathrm{AP}}+S_{P}=S_{↑}+S_{↓}$$

and

(8) $$S_{\mathrm{AP}}-S_{P}=\beta \left(S_{↓}-S_{↑}\right),$$

where $\beta =\left(\rho_{↓}-\rho_{↑}\right)/\left(\rho_{↓}+\rho_{↑}\right)$ denotes the spin asymmetry coefficient for resistivity. Combining Equations (7) and (8), the spin-dependent Seebeck coefficients, $S_{↑}$ and $S_{↓}$ can be expressed as follows [13]:

(9) $$S_{↑}=\frac{1}{2}\left[S_{\mathrm{AP}}\left(1-\beta^{-1}\right)+S_{P}\left(1+\beta^{-1}\right)\right],$$

(10) $$S_{↓}=\frac{1}{2}\left[S_{\mathrm{AP}}\left(1+\beta^{-1}\right)+S_{P}\left(1-\beta^{-1}\right)\right].$$

From Equations (9) and (10), it can be easily deduced that $S_{↑}=S_{P}$ and $S_{↓}=S_{\mathrm{AP}}$ in the limits of an extremely large MR ratio (β→1).

The temperature dependencies of $S_{\mathrm{AP}}$, $S_{P}$, $S_{↑}$ and $S_{↓}$ are shown in Figure 9b–d for the Co/Cu, Co${}_{50}$Ni${}_{50}$/Cu and Ni${}_{80}$Fe${}_{20}$/Cu multilayered NW networks. Below RT, the various Seebeck coefficients of all three samples decrease almost linearly with decreasing temperature, which is another indicative of the dominance of diffusion thermopower. The deviation from the linear regime in Figure 9b–d can be ascribed to other possible contribution to the thermopower such as magnon-drag. The estimated values at RT of $S_{↑}$ and $S_{↓}$ are reported in Table 3, using $\beta ={\mathrm{MR}}^{1/2}$ in Equations (9) and (10). The values of $S_{↑}$ and $S_{↓}$ for Co/Cu NWs are similar to those previously reported in bulk Co ($S_{↑}=-30$μV/K and $S_{↓}=-12$μV/K) [46]. In contrast, the value for $\Delta S=S_{↑}-S_{↓}$ for Co/Cu, Co${}_{50}$Ni${}_{50}$/Cu and Ni${}_{80}$Fe${}_{20}$/Cu NWs shown in Table 3, in the range of −8 to −12 μV/K [13,14,22], are much larger than the ones of −1.8 μV/K and −3.8 μV/K to −4.5 μV/K extracted from measurements performed on Co/Cu/Co nanopillar spin valve and Ni${}_{80}$Fe${}_{20}$/Cu/Ni${}_{80}$Fe${}_{20}$ nanopillar and lateral spin devices, respectively, using a 3D finite-element model [6,68]. The largest spin-dependent Seebeck coefficient is found in Ni${}_{80}$Fe${}_{20}$/Cu NWs, reaching −12.3 μV/K [22]. From the estimated values of $S_{↑}$ and $S_{↓}$, the RT spin asymmetry for Seebeck coefficients $\eta =\left(S_{↓}-S_{↑}\right)/\left(S_{↓}+S_{↑}\right)$ has been estimated, and are reported in Table 3 for the interconnected Co/Cu, Co${}_{50}$Ni${}_{50}$/Cu and Ni${}_{80}$Fe${}_{20}$/Cu NWs, respectively. It is also found that the η value found for the crossed Co/Cu NW network is consistent with the values previously reported for parallel Co/Cu NWs [64].

In the limit of MR $=\beta^{2}$, the magnetothermopower can also be expressed as [13]:

(11) $$\mathrm{MTP}=\frac{2\beta \eta }{1+\beta \eta }.$$

Considering first the case |βη|≪ 1, this leads to MTP/MR≈2η/β, which means that enhancement of the MTP ratio compared to the corresponding MR ratio is expected if 2|η|>|β|. The amplitudes of the MR and MTP effects for the Co/Cu, Co${}_{50}$Ni${}_{50}$/Cu and Ni${}_{80}$Fe${}_{20}$/Cu NW networks studied have been found comparable at RT, with 2|η|≈|β| as seen in Table 3. Similarities between the amplitudes of the MR and MTP at RT were already observed in arrays of parallel Co/Cu NWs [64,69,69] and Co/Cu CIP multilayers [57,70]. However, the physical reason of this is unclear. In contrast to the other FM/Cu NW networks, the Ni/Cu system shows a much larger MTP ratio than its corresponding MR ratio, as seen in Figure 7d. This significant boost of the MTP, compared to MR may indicate an underlying large η coefficient due to contrasting $S_{↑}$ and $S_{↓}$ values, while β is expected to be small in Ni. This is consistent with the values of $S_{↑}=$ −43 μV/K and $S_{↓}=$ 20 μV/K reported by Cadeville and Roussell [46], leading to η≈ −3. Furthermore, from Equation (11), infinitely large MTP effect is expected when the product βη tends to −1. While |β|<1, |η|>1 can be reached if $S_{↑}$ and $S_{↓}$ exhibit opposite signs. Therefore, the fabrication of multilayered NWs with appropriate magnetic layer composition should make it possible to fine-tune the power factor of thermoelectric energy conversion with an external magnetic field. The limit case of an infinite MTP ratio underlies that the Seebeck coefficient obtained in the antiparallel state $S_{\mathrm{AP}}$ reaches 0. Indeed, the Seebeck coefficients in the antiparallel and parallel states can be expressed as

(12) $$S_{\mathrm{AP}}=\frac{S_{↑}+S_{↓}}{2}\left(1+\beta \eta \right)$$

and

(13) $$S_{P}=\frac{S_{↑}+S_{↓}}{2}\left(1-\beta \eta \right).$$

For βη= −1, this yields $S_{\mathrm{AP}}=$ 0 and $S_{P}=S_{↑}+S_{↓}$. As a consequence, for a practice device, Seebeck coefficients for spin up and spin down electrons of opposite sign and with largely asymmetrical amplitudes are required. This would lead to a system where at zero magnetic field, no thermoelectric current is generated, while under an applied external magnetic field, a large thermoelectric current is generated. It should be noted that the limit case βη= 1 yields the opposite scenario where $S_{\mathrm{AP}}=S_{↑}+S_{↓}$ and $S_{P}=0$. In that case, a thermoelectric current is generated in absence of magnetic fields, which vanishes under an applied external magnetic field. The case βη→ 1 also requires |η|>1 and therefore Seebeck coefficients for spin up and spin down with opposite signs. Moreover, Equation (7) indicates that the more different their amplitudes, the larger the $S_{\mathrm{AP}}$ value, which is required for practical applications. In this context, the Ni/Cu system is expected to be a good potential candidate for the observation of highly enhanced MTP effect, since β>0 and, according to Cadeville and Roussel [46], η≈ −3. Experimental challenge lies with making interconnected Ni/Cu NW networks with larger magneto-transport properties.

Interestingly, it can be shown that the diffusion thermopower of a ferromagnetic homogeneous NW network can be expressed as

(14) $$S_{\mathrm{FM}}=\frac{S_{↑}\alpha +S_{↓}}{\alpha +1}.$$

Therefore, because α≫1 is expected for Co, the Seebeck coefficient obtained for homogeneous Co NW networks (−28 μV/K, see Table 1) is consistent with $S_{\mathrm{Co}}\approx S_{\mathrm{Co},↑}$. Moreover, the addition of a very small amount of Cr in Co is expected to induce α≪1. As a consequence, the Seebeck coefficient of dilute CoCr alloys can be approximated by $S_{{\mathrm{Co}}_{99}{\mathrm{Cr}}_{1}}\approx S_{\mathrm{Co},↓}$, assuming that the very small amount of Cr does not significantly impact the Seebeck coefficient of spin down electron, which is also in good agreement with the value reported for ${\mathrm{Co}}_{99}{\mathrm{Cr}}_{1}$ (−12 μV/K). In contrast, because α≈1 is expected for Ni, Equation (14) leads to $S_{\mathrm{Ni}}=\left(S_{↑}+S_{↓}\right)/2$. The values of $S_{\mathrm{Ni}}\approx$ −20 μV/K is found in good agreement with the Seebeck coefficients for spin up and spin down electrons reported by Cadeville and Roussell ($S_{↑}\approx -43$μV/K and $S_{↓}\approx$ +20μV/K [46]). Moreover, the addition of diluted Cr impurities in Ni is also expected to induce α≪1. Therefore, the Seebeck coefficient of dilute NiCr alloys is expected to tend towards the value of $S_{\mathrm{Ni},↓}$. The positive values obtained in dilute NiCr alloys is in good agreement with the positive value of $S_{\mathrm{Ni},↓}$ reported by Cadeville and Roussell [46] as shown in Figure 4c. This is another indication that Ni may exhibit a probable spin-dependent Seebeck coefficients of opposite sign.

## 4. Conclusions

This research provides a simple and cost-effective pathway to fabricate highly efficient and large-scale nanowire-based thermoelectric films meeting key requirements for electrical, thermal and mechanical stability. Flexible and planar interconnected nanowire networks allows for both p- and n-type thermoelectric modules with large room-temperature Seebeck coefficient and power factors. Since there is no sample size limitation, this fabrication method is expandable into network films with much larger dimensions. A practical planar thermoelectric cooler made of flexible and shapeable thermoelectric modules consisting of stacked nanowire network films that are connected electrically in series and thermally in parallel can be easily obtained. Furthermore, an unexpected high value of the magneto-thermopower effect compared with that of the corresponding magnetoresistance effect has been observed in Ni crossed nanowires, in agreement with previous studies on Ni nanowires.

Embedded nanowire networks in porous polymer films are also perspective materials for spin caloritronics applications. Centimetre-scale interconnected network films made of multilayered nanowires show giant magnetoresistance and giant magneto-thermoelectric effects together with large and magnetically modulated room-temperature thermoelectric power factor up to 5 mW/K${}^{2}$m. These macroscopic nanowire networks also enable the direct extraction of key material parameters for spin caloritronics such as spin-dependent Seebeck coefficients. Spin-dependent Seebeck coefficients up to −12.3 μV/K have been obtained at room temperature in Ni${}_{80}$Fe${}_{20}$/Cu multilayered nanowire networks. Moreover, a large enhancement of the magneto-thermopower ratio compared to the corresponding magnetoresistance ratio of about four times larger at room temperature has been measured in Ni/Cu nanowire networks, potentially indicating high spin-dependent Seebeck coefficients in Ni, consistent with previous studies. These results open an exciting and a promising pathway for the next generation of flexible and lightweight thermoelectric devices exploiting the spin degree of freedom and the realization of magnetic thermal switch for heat management. In addition, flexible thermoelectric films based on macroscopic networks of interconnected nanowires can also be used in applications for devices with low energy requirements where the heat input is essentially free, as in the case of waste heat.

## Figures and Tables

**Table 1:** Room-temperature Seebeck coefficient *S*, resistivity ρ, power factor (PF), figure of merit ZT, magnetoresistance ratio (MR) and magnetothermopower ratio (MTP) of interconnected homogeneous nanowire networks made of ferromagnetic metals and alloys.

	*S* (μV/K)	ρ (μΩcm)	PF (mW/K${}^{2}$m)	ZT (-)	MR (%)	MTP (%)
Co	−28.0	7.1	11.0	3.2·10${}^{-2}$	1.1	−1.1
Fe	+15.0	12.8	1.8	9.1·10${}^{-3}$	0.2	-
Ni	−19.6	9.1	4.2	1.6·10${}^{-2}$	1.6	−6.0
Co${}_{50}$Ni${}_{50}$	−18.3	15.4	2.2	1.4·10${}^{-2}$	2.9	−3.8
Ni${}_{90}$Fe${}_{10}$	−34.4	18.6	6.3	4.8·10${}^{-2}$	3.3	−2.1
Ni${}_{80}$Fe${}_{20}$	−36.9	25.0	5.4	5.6·10${}^{-2}$	2.4	−1.5
Ni${}_{70}$Fe${}_{30}$	−40.2	32.5	5.0	6.6·10${}^{-2}$	1.5	−1.3
Ni${}_{60}$Fe${}_{40}$	−46.0	42.4	5.0	8.6·10${}^{-2}$	0.7	−0.8
Ni${}_{96}$Cr${}_{4}$	+15.5	27.3	0.9	9.8·10${}^{-3}$	0.1	-

**Table 2:** Room-temperature Seebeck coefficient $S_{P}$, resistivity $\rho_{P}$, power factor PF${}_{P}$ and figure of merit $ZT_{P}$ obtained in the saturated state for interconnected multilayered nanowire networks made of a stack of successive ferromagnetic metal and Cu layers, as well as their magnetoresistance ratio MR and magneto-thermopower ratio MTP.

	$S_{P}$ (μV/K)	$\rho_{P}$ (μΩcm)	${\mathrm{PF}}_{P}$ (mW/K${}^{2}$m)	${\left(\mathrm{ZT}\right)}_{P}$	MR (%)	MTP (%)
Co/Cu	−19.9	8.7	4.6	1.6·10${}^{-2}$	24.7	−25.1
Co${}_{50}$Ni${}_{50}$/Cu	−21.6	10.2	4.6	1.9·10${}^{-2}$	30.2	−33.7
Ni${}_{80}$Fe${}_{20}$/Cu	−24.8	15.3	4.0	2.5·10${}^{-2}$	17.1	−25.8
Ni/Cu	−10.2	19.8	0.5	0.4·10${}^{-2}$	0.8	−3.7

**Table 3:** Room-temperature spin-dependent Seebeck coefficients, $S_{↑}$ and $S_{↓}$, of the FM/Cu nanowire networks with FM = Co, Co${}_{50}$Ni${}_{50}$, Ni${}_{80}$Fe${}_{20}$ an Ni, along with $\Delta S=S_{↑}-S_{↓}$ and the spin asymmetry coefficients for resistivity β and Seebeck coefficient η.

	$S_{↑}$ (μV/K)	$S_{↓}$ (μV/K)	ΔS (μV/K)	β (-)	η (-)
Co/Cu	−23.6	−15.1	−8.5	0.50	−0.22
Co${}_{50}$Ni${}_{50}$/Cu	−23.9	−13.9	−10.0	0.55	−0.26
Ni${}_{80}$Fe${}_{20}$/Cu	−28.4	−16.1	−12.3	0.41	−0.28
